# Books and Book Reviews

**Published:** 1887-07

**Authors:** 


### BOOKS AND BOOK REVIEWS.

A PRACTICAL TREATISE ON OBSTETRICS. VOL. IV. Obstetric Operations. The Pathology of the Puerperium. By A. CHARPENTIER, M.D., Paris. Illustrated with lithographic plates and wood engravings. This is also Vol. IV. of the "*Cyclopedia of Obstetrics and Gynecology*" (12 vols.), issued monthly during 1887. NEW YORK: WILLIAM WOOD & COMPANY. CHICAGO: W. T. KEENER.

Anything which even approaches in its nature a complete and critical review of so large and important a work as Charpentier's Obstetrics, is beyond the province of this journal, and general statements in this instance must suffice. Favorable mention has already been made of the preceding three volumes of this translation. This concluding volume only deepens the impression made by the former numbers.

The editorial notes have been a very important and acceptable addition to the text throughout the work, and this is strikingly true of volume IV. They make the book representative of the best opinion up to date, and save it from the charge that the original is already a little behind the times. This is one of the few books of which it may be truthfully said that no specialist interested in the subject, and no general practitioner can be without, and yet be fully posted in his line of work. One feature still deserves unmeasured condemnation: another edition with the present cuts would be unpardonable.

HANDBOOK OF MATERIA MEDICA, PHARMACY, AND THERAPEUTICS, including the Physiological Action of Drugs, the special Therapeutics of Disease, official and extemporaneous Pharmacy, and Minute Directions for Prescription-writing. By SAMUEL O. L. POTTER, M. A., M. D., Professor of the Theory and Practice of Medicine in the Cooper Medical College of San Francisco: Author of *Quiz-Compends* of Anatomy and Materia Medica; *An Index of Comparative Therapeutics*, and *A Study of Speech and its Defects*. Late A. A. Surgeon, U. S. Army. Pp. xx, 828. PHILADELPHIA: P. BLAKISTON, SON & CO. CHICAGO: W. T. KEENER.

As announced in the preface, the intention has been to produce a book which would embrace, in a single volume, the essentials of materia medida and therapeutics, such instruction in pharmacy as every physician should possess, and minute directions for prescription-writing.

Unfortunately the general style of the author's *Quiz-Compend* of Materia Medica is retained in this hand-book, which can be regarded only as a voluminous compendium of pharmacological, therapeutic and pharmaceutical data, in which, for the sake of brevity, all experimental processes and methods by which stated results have been ascertained, are omitted. For instance, the physiological action of digitalis on the heart is epitomized, merely, within a dozen lines, a treatment of the subject which is not commensurate with its importance.

As a sole reliance, the work is inadequate to the needs of students, because, like all compends, it appeals exclusively to the memory and furnishes no scope for assistance by exercise of the reasoning faculties.

Part II, treating briefly of pharmacy and prescription-writing, is a commendable addition.

Part III, pertaining to special therapeutics, is composed of an alphabetical list of diseases with a summary of the therapeutic indications in each. It is the customary index of diseases, extended and enlarged.

A section is appended embracing tables of Latin words and phrases used in prescription-writing, hypodermic formulæ, formulæ of patent medicines, the treatment of poisons, differential diagnosis notes on temperatures in disease, obstetrical memoranda, the treatment of asphyxia, as in drowning, clinical examination of urine, extracts from the code of ethics, etc., some of which miscellaneous information is *hors de propos*, but all of which is of a most useful and instructive nature.

As the work is systematically arranged, its statements reliable, and the index perfect, it will be serviceable for ready reference and review.

EVACUANT MEDICATION: Cathartics and Emetics. By HENRY M. FIELD, M.D. Pp. vi. and 288. PHILADELPHIA: P. BLAKISTON, SON & CO. CHICAGO: W. T. KEENER. 1887.

There are few subjects in the domain of internal medicine, usually considered as of minor interest, which are really of more practical importance than this. In our medical colleges it rarely, if ever, receives systematic attention, and the standard text-books do not give it comprehensive treatment. The recent medical graduate, if a conscientious prescriber, is hardly ever so much at a loss as in the choice of a cathartic for individual cases, and in no other aspect of his work does the older practitioner have so great an advantage over him. The author's remark, that "popular and professional abuse of cathartics was never so great as now," is amply supported by the larger number and expensiveness of the advertisements of cathartic medicines. The author treats his subject in a systematic, thorough and very satisfactory manner, as the table of contents will demonstrate. Its divisions are : Introductory. EVACUANTS—No 1. CATHARTICS; Chologogues, Mineral Cathartics, Differential Therapeusis, Cathartic Medication, General Principles. EVACUANTS—No. 2. EMETICS; Topical or Irritant Emetics, Emetic Medication, Arrest of Vomiting, and an Index which is complete and satisfactory.

A TEXT-BOOK OF HUMAN PHYSIOLOGY, INCLUDING HISTOLOGY AND MICROSCOPICAL ANATOMY, WITH SPECIAL REFERENCE TO THE REQUIREMENTS OF PRACTICAL MEDICINE. *By* DR. L. LANDOIS, *Second American edition, translated from the fifth German edition, with additions. By* WILLIAM STERLING, M. D., Sc. D. *Pp. xxxix 922 ; 583 illustrations. Philadelphia :* P. BLAKISTON, SON & CO. *1886. Chicago :* W. T. KEENER.

This second translation of Professor Landois' "Lehrbuch der Physiologie des Menschen" is, in many respects, an improvement upon the first. The same general arrangement has been preserved, but some of the chapters have been largely re-written, and for the most part improved. The translator has done his work well, having reached a happy medium, neither very closely imitating the text nor departing too widely from it. The work is not easy reading, the style being somewhat disjointed—a defect that is more apparent in the original than in the translation, and one that was unavoidable in crowding this amount of matter into a single volume of this size. A large number of authors have been consulted, and their names appear in the text. In fact, for almost every statement the authority is given, but not the reference. This is an unfortunate omission, as they could easily have been added in foot notes without materially increasing the size of the book. The work is too voluminous for the student of medicine, and for occasional reference in the library of the physician such foot notes would have been invaluable.

The arrangement of the work is admirable, being divided into sections, sub-sections and paragraphs, each one designated by double-face types; thus making it possible to pick out at a glance the precise subject on which information is sought, and rendering a reference to the index scarcely necessary.

For the use of the practicing physician it is one of the best books in the English language ; for those just beginning their studies a smaller work would be better suited.

THE PRINCIPLES AND PRACTICE OF OPERATIVE SURGERY. *By* STEPHEN SMITH, A. M., M. D., *Professor of Clinical Surgery in the University of the City of New York, Surgeon to Bellevue Hospital, etc., etc. New and thoroughly revised edition. Pp. xxxii and 877. Philadelphia:* LEA BROTHERS & CO. *Chicago:* A. C. MCCLURG & CO. *1887.*

This work was first published in 1879, and has had eight distinct issues since ; but this, being the first revised, is really the second edition. The revision has been thorough and complete, and it brings the book fully up with the latest approved measures in operative surgery. In preparing the text upon antiseptics and their employment, the author says that he has drawn freely upon the works of Cheyne and Von Hacker. This condensation has been admirably made, and the author's directions can be understood by any surgeon. It is gratifying to have a text-book in English on operative surgery which, without reserve, condemns as inexcusable all operative surgery which is not strictly aseptic. This edition is decidedly superior to its ambitious and excellent rival which has recently appeared, and which was reviewed in this journal.

DISEASES OF WOMEN. A hand-book for Physicians and Students. By DR. F. WINCKEL, of Munich. Authorized translation by J. H. WILLIAMSON, M. D., under the supervision and with an introduction by THEOPHILUS PARVIN, M. D. PHILADELPHIA: P. BLAKISTON, SON & CO. CHICAGO: W. T. KEENER. 1887.

This translation of Professor Winckel's handbook is a welcome addition to the treatises upon the subject in the English language. The standing of the author of the original is so well assured among his colleagues in Germany that he needs no eulogy here. The teaching of the book is eminently conservative, and in this reflects the growing tendency among the Germans. The treatment of the subjects discussed is clear, practical and thorough. The translation is excellent and the letter press very satisfactory. The book deserves a large sale among general practitioners and students.

